# Understanding the null‐to‐small association between increased macroeconomic growth and reducing child undernutrition in India: role of development expenditures and poverty alleviation

**DOI:** 10.1111/mcn.12256

**Published:** 2016-05-17

**Authors:** William Joe, Ramaprasad Rajaram, S. V. Subramanian

**Affiliations:** ^1^ Institute of Economic Growth Delhi University Enclave Delhi 110 007 India; ^2^ Athena Infonomics Chennai India; ^3^ Faculty, Institute of Mental Health, Social Sciences and Trans‐disciplinary Research Chennai India; ^4^ Harvard Center for Population and Development Studies Cambridge, MA USA; ^5^ Department of Social and Behavioral Sciences Harvard T.H. Chan School of Public Health Boston Massachusetts USA

**Keywords:** stunting, undernutrition, economic growth, poverty, development expenditure, growth‐mediated strategy, support‐led strategy, India

## Abstract

Empirical evidence suggests that macroeconomic growth in India is not correlated with any substantial reductions in the prevalence of child undernutrition over time. This study investigates the two commonly hypothesized pathways through which macroeconomic growth is expected to reduce child undernutrition: (1) an increase in public developmental expenditure and (2) a reduction in aggregate income‐poverty levels. For the anthropometric data on children, we draw on the data from two cross‐sectional waves of National Family Health Survey conducted in 1992–1993 and 2005–2006, while the data for per capita net state domestic product and per capita public spending on developmental expenditure and headcount ratio of poverty were obtained from the Reserve Bank of India and the Government of India expert committee reports. We find that between 1992–1993 and 2005–2006, state‐level macroeconomic growth was not associated with any substantial increases in public development expenditure or substantial reductions in poverty at the aggregate level. Furthermore, the association between changes in public development expenditure or aggregate poverty and changes in undernutrition was small. In summary, it appears that the inability of macroeconomic growth to translate into reductions in child undernutrition in India is likely a consequence of the macroeconomic growth not translating into substantial investments in development expenditure that could matter for children's nutritional status and neither did it substantially improve incomes of the poor, a group where undernutrition is also the highest. The findings here build a case to advocate a ‘support‐led’ strategy for reducing undernutrition rather than simply relying on a ‘growth‐mediated’ strategy.

**Key messages**

- Increases in macroeconomic growth have not been accompanied by substantial increases in public developmental spending or reduction in aggregate poverty headcount ratio in India.
- Association between increases in public development expenditure or poverty headcount ratios and changes in child undernutrition, in particular, child stunting, is small to null.
- Reducing the burden of undernutrition in India cannot be accomplished solely relying on a growth‐mediated strategy, and a concerted support‐led strategy is required.

## Introduction

It is clear that while there exists an inverse ecological association between *levels* of per capita income and prevalence of child undernutrition (Smith & Haddad 2002; Haddad *et al.* 2003; Smith & Haddad 2015), results from studies examining the association between *changes* in per capita income and child undernutrition have been null to small, both across countries (Heltberg 2009; Vollmer *et al.* 2014) and across states in India (Subramanyam *et al.* 2011). Furthermore, regardless of whether ecological studies are cross‐sectional or repeated cross‐sectional, they cannot quantify the association between aggregate macroeconomic growth and the risk of undernutrition at the child level (Subramanyam *et al.* 2011; Vollmer *et al.* 2014), which suggests the need for more studies to investigate this relationship in a multilevel framework. The null‐to‐small association between macroeconomic growth and reductions in child undernutrition in India conducted within a multilevel framework (Subramanyam *et al.* 2011) begs further investigation, given the overwhelming reliance on a ‘growth‐mediated’ strategy to improving population health (Dreze & Sen 1989; Dreze & Sen 2013; Subramanian *et al*. 2016). It should be acknowledged that even though the association between increases in macroeconomic growth measured at the state level and decreases in the likelihood of child undernutrition is small, there does exist an inverse association between household wealth measured through asset index at the micro level and the likelihood of child undernutrition (Subramanyam *et al.* 2011). Some caution is necessary to naively interpret such a gradient as evidence of the effect of macroeconomic growth, especially because the association between macroeconomic growth and likelihood of child undernutrition in India was found to be small‐to‐null even without adjusting for micro/household wealth (Subramanyam *et al.* 2011). Furthermore, it has also been shown that household wealth index growth and standard measures of economic growth (often based on consumption‐based income measures) are not correlated and remain largely unaffected by trends in macroeconomic performance (2013a, 2013b).

Consequently, employing ecological and multilevel analysis, we aim to advance the literature by exploring the role of two primary pathways through which increases in macroeconomic growth could substantially translate into reducing the burden of child undernutrition. First, increased macroeconomic growth could potentially lead to increased public development expenditure (especially in social and health sectors) that in turn can lead to reductions in child undernutrition. Second, increased macroeconomic growth can also directly reduce child undernutrition by improving the material standards of living of the majority of the population, i.e. raising income levels and/or substantially reducing number of people living in poverty. In the absence of substantial investments in public development expenditures that matter for health or substantial reductions in poverty, it will not be surprising for macroeconomic growth not to be associated with the reductions in child undernutrition in India. Hence, in this study, we investigate (1) whether macroeconomic growth led to increases in public development expenditure, and reductions in incidence of poverty across Indian states, and (2) whether changes in public development expenditure and poverty incidence were associated with reductions in undernutrition in children under the age of 3 years. If these two effects are not significant, then one might hypothesize that not only macroeconomic growth in India is not associated with undernutrition but also the association between ‘changes’ in poverty and public development expenditure with undernutrition is negligible.

## Methods

### Data

The data for this analysis are obtained from the National Family Health Surveys (NFHS) of India. The NFHS is part of the Demographic and Health Surveys programme that provides technical assistance to more than 300 surveys on health and population issues in over 90 developing countries (http://www.dhsprogram.com). NFHS‐3 (2005–2006), the third in the series of these national surveys, was preceded by NFHS‐2 in 1998–1999 and NFHS‐1 in 1992–1993. With a focus on maternal and child health, NFHS adopts a multi‐stage, systematic and stratified sampling design to provide national and sub‐national information on fertility, family planning, maternal and child health, gender, HIV/AIDS, malaria and nutrition (see IIPS 1995; IIPS and Macro International, 2007 for details on data collection and methodologies in the survey). In our analysis, owing to unavailability of comparable data across the three surveys, we could use only the first (1992–1993) and the third (2005–2006) NFHS surveys. NFHS 1992–1993 and NFHS 2005–2006 cover 48 959 and 51 555 children (including 3680 and 2876 death cases), respectively, and we undertook the following step‐wise filtration process to arrive at our final sample for analysis. In the first step, we dropped the cases with missing information (including death cases) on height or weight of the children (19 692 and 7685 cases in NFHS 1992–1993 and 2005–2006, respectively). To ensure comparability with previous research, we restricted the analysis to children aged below 3 years, which leads to a further reduction in the sample size by 6935, and 17 592 cases, respectively. It may be noted that under the first wave of NFHS, anthropometric information was not ascertained from certain sample regions and states. Specifically, the survey did not ascertain information on children's height‐for‐age in the states of Andhra Pradesh, Himachal Pradesh, Madhya Pradesh, Tamil Nadu and West Bengal. Because our analysis focuses on state‐specific dynamics (as explained in detail later), to maintain consistency across the two surveys, we excluded these states in the 2005–2006 survey. Due to unavailability of information regarding public developmental expenditure, which is one of the key variables of interest, we had to exclude the states of Mizoram, Nagaland and Tripura from the two waves. Also, in the year 2000, two states – Uttar Pradesh and Bihar – were bifurcated; therefore, to maintain comparability across the two surveys (one pre 2000 and one post), we treated the two states as undivided by combining the new states Jharkhand and Uttarakhand with their corresponding parent states – Bihar and Uttar Pradesh. We finally arrived at the following pooled sample: stunting – 37 256 cases, underweight – 38 817 cases and wasting – 37 073 cases. We also include the following covariates for analysis from the NFHS: age, sex (value of 1 for boy and 0 for girl), birth order of the child; maternal age at child birth, maternal and paternal education, marital status (value of 1 if married and 0 otherwise); social group – caste and religion (explained later); and household asset‐based wealth index ranking, and place of residence (value of 1 for rural and 0 otherwise).

Recently, the Government of India and UNICEF have released the results of Rapid Survey on Children (RSOC) conducted during 2013–2014 to strengthen the data system on children and women, based on a nationwide household *cum* facility‐based survey in 28 states and Delhi. This survey has a focus on the well‐being of children below 6 years and their mothers covering aspects of child health and development maternal health and health care and school/college attendance among persons aged 5–24 years. However, it may be noted that the unit level data from the RSOC survey is unavailable (at the time of writing the paper) in the public domain to facilitate an individual level analysis. Therefore, we only present some inferences based on ecological analysis of the RSOC data as Supporting Information tables.

Additional data to characterize the state level per capita net state domestic product (PCNSDP), public developmental expenditure [per capita state developmental expenditure (PCDE)] and poverty headcount ratio (HCR) are based on the official sources. The information on PCNSDP is prepared by the Central Statistical Organization of the Ministry of Statistics and Program Implementation, Government of India. The information on PCNSDP is also made available in the Reserve Bank of India, which is used in our analysis, for the years 1993–1994, 2005–2006 and 2013–2014 corresponding to the two NFHS waves and the RSOC survey. The information on PCDE is compiled by the Reserve Bank of India and is provided in its annual publication on state finances (available at https://www.rbi.org.in). Information on poverty HCR – defined as percentage of population below the official poverty line – is obtained from the report of the Government of India (Government of India 2009). These estimates are based on the data from the private household consumer expenditure and collected by the National Sample Survey Organization, and we used the state‐level data for these variables. As mentioned earlier, owing to unavailability of comparable HCR estimates for the year 1998–1999, we could not include the NFHS second wave information in our pooled repeated cross‐sectional data analysis. We have also used poverty estimates from the private consumer expenditure survey data for 2011–2012 to facilitate an ecological analysis using the RSOC data on child undernutrition across Indian states.

### Outcomes, exposure and covariates

Undernutrition among children is based on anthropometric data (physical indices) made available through NFHS (we also use data from the RSOC to verify our results). The anthropometric measures describe nutritional status of children with respect to three dimensions: height‐for‐age (stunting), height‐for‐weight (wasting) and weight‐for‐age (underweight). While our focus is predominantly on chronic undernutrition – stunting, we also verify our findings with the other two measures as well. A child is considered stunted, wasted or underweight if he or she falls two standard deviations below the median score for children of the same age and gender in the reference population on their respective anthropometric scores. The median score of the reference population is based on an internationally accepted World Health Organization Child Growth Standards, which is applied in both the NFHS waves and helps identify if a child is undernourished. The computation of *z*‐score is performed using the stata user‐written programme *zscore06* by Leroy (2011). It may be noted that unlike WHO's *igrowup* programme, none of the *z*‐scores are calculated if child age is missing. For stata commands related to the calculation of anthropometric *z*‐scores using the WHO (2006) child growth standards, see http://www.ifpri.org/staffprofile/jef-leroy.

Per capita net state domestic product, PCDE and state poverty HCR are the key socio‐economic indicators of interest. PCNSDP is expressed in Indian Rupees (INR) and is a fundamental indicator of a state's income and economic progress. To adjust for price variations over the years, PCNSDP is expressed in 2004–2005 constant prices and normalized to 5000 to get the estimates in units of INR 5000. For 2005, the PCNSDP of the two new states (Uttarakhand and Jharkhand) was combined with its parent state (Uttar Pradesh and Bihar, respectively) by using the information on population size and PCNSDP of these states.

The PCDE (in INR) is expressed in 2004–2005 prices and normalized to 2000 to get estimates in units of INR 2000. Similar to PCNSDP, for the year 2005, the PCDE of new states (Uttarakhand and Jharkhand) was combined with its parent state (Uttar Pradesh and Bihar, respectively) by using the information on population size and PCNSDP of these states. Expenditures of the state governments in India are categorized into developmental and non‐developmental expenditure, and developmental expenditure of the state is defined as revenue and capital expenditure on social and economic services. The resources for the same are also generated through contribution of the central government or by means of loans and advances to the state. Developmental expenditure on social services generally covers areas such as health, education, water supply, sanitation, housing, social security and welfare of marginalized and vulnerable subgroups such as the Scheduled Castes and Tribes – SC/ST. Under economic services, developmental spending is largely on agriculture and allied activities, rural development, irrigation, energy, transport and communication.

For analytical purposes, maternal age at child birth was divided into five categories: less than 17 years (reference category), 17–19, 20–24, 25–29 and more than 29 years. Mother's marital status was scored 1 if she was living with her husband and 0 (i.e. single) if she was widowed, divorced or separated. Education of mother and father was categorized using the customary classification in the Indian educational system as follows: illiterate – no formal schooling (reference category), primary – up to 5 years of schooling, secondary – up to 10 years of schooling and higher – more than 10 years of schooling. Social group affiliation is categorized as scheduled caste, scheduled tribe and others (reference category). Typically, the ‘others’ subgroup (reference category) – for caste – is considered to be relatively advantaged in terms of general socio‐economic conditions: in fact, there are specific legal, constitutional and policy provisions for promotion of social and economic welfare among the scheduled caste and scheduled tribe population. Further, households were classified based on religious affiliation (Hinduism, Islam and others). To account for economic status of the households, we used the asset‐based wealth index derived through principal component analysis (Filmer & Pritchett 2001; IIPS Macro‐International 2007). Descriptive statistics for all the key variables are provided in Table S1.

### Analysis

We use alternative model specifications to understand how macroeconomic growth translated to increased PCDE and poverty reduction, and in turn, how the latter two economic development indicators influenced child undernutrition across Indian states. We employed two types of analysis: an aggregate cross‐sectional analysis at the state level for each time period and an aggregate repeated cross‐sectional analysis (using states as a fixed effect). While the former estimates the association between two variables at a given level, the latter estimates whether change in one variable is associated with a change in another. The latter specification is the one that we use for interpretation.

Further, we also estimated multilevel logistic regression models with a log link function to exploit the latent association between economic variables at aggregate levels and undernutrition at the individual level. The model estimation is based on penalized quasi‐likelihood procedures with first‐order Taylor linearization (Rasbash *et al.* 2009). The results are presented both for unadjusted model (not controlled for the entire set of potential covariates but clustered at the primary sampling unit level) and the fully adjusted models (which includes controls for set of socio‐economic variables as well). We report the odds ratios along with 95% confidence intervals.

In the modelling exercise we adopt a stepwise approach, wherein first, we estimate the association of each indicator of child undernutrition with PCNSDP, PCDE and HCR separately, adjusted for age and sex of the child; subsequently, all the socio‐economic covariates are included to present results from the fully adjusted model. As a general rule, wherever we employ time as the predominant unit of analysis, i.e. change in change models, we control for state‐fixed effects. While the main text presents estimates based on unweighted regressions, we also perform the analysis using specified sampling weights and arrive at similar conclusions. Data management was performed in Stata 13.0 version, whereas the multilevel models were implemented using *runmlwin* programme developed for the use of MLwiN (2.31 version) statistical software within Stata (Leckie and Charlton 2013, StataCorp 2013).

Given our focus on chronic undernutrition, the results are discussed in detail for stunting, while results for underweight and wasting are presented in Supporting Information tables.

## Results

### Descriptive patterns

Per capita net state domestic product, PCDE and HCR varied widely across the Indian states between 1993 and 2005 (Table 1). Bihar and Goa had the lowest and highest PCNSDP, respectively, at both time periods. Gujarat registered the highest average annual PCNSDP growth of 7.5% over the period, while Assam registered the lowest: 1.4%. Interestingly, in 1993, the PCDE spending in Bihar was about one‐sixth of PCDE in Goa, and this ratio further decreased to one‐tenth in 2005. In 1993, six states had poverty HCR exceeding 50%, and despite macroeconomic growth, Odisha and Bihar continued to have more than half of its population below the poverty line: 57% and 54%, respectively.

**Table 1:** Statewise prevalence of developmental indicators, Indian States 1992–1993 and 2005–2006

States	Stunting (%)		PCNSDP (INR)		PCDE (INR)		Poverty HCR (%)	
	1993	2005	1993	2005	1993	2005	1993	2005
Arunachal Pradesh	56.5	34.3	18 910	26 759	7879	12 512	55	31
Assam	56.0	41.0	14 601	17 050	1995	2439	52	34
Bihar	59.4	48.9	6257	7749	1157	1329	61	54
Delhi	47.7	43.0	41 659	69 128	1112	4839	16	13
Goa	35.3	26.2	46 804	80 844	6945	12 546	21	25
Gujarat	50.0	48.6	19 060	36 102	2387	3772	38	32
Haryana	50.2	43.5	23 919	40 627	2450	3916	36	24
Jammu and Kashmir	44.2	32.7	17 262	22 406	3932	7953	26	13
Karnataka	47.2	40.8	17 034	29 295	2372	3808	50	33
Kerala	32.8	27.4	18 897	34 837	2149	3099	31	20
Maharashtra	46.4	43.9	24 918	40 671	2456	4039	48	38
Manipur	31.9	29.3	14 204	19 479	2788	6309	65	38
Meghalaya	53.3	42.7	15 057	25 642	4118	5179	35	16
Odisha	50.3	43.7	12 009	18 194	1674	1892	59	57
Punjab	43.7	36.5	26 096	34 096	2541	3116	22	21
Rajasthan	46.1	39.1	12 256	19 445	1896	2683	38	34
Uttar Pradesh	59.2	51.6	10 815	13 445	1378	1687	48	41

Stunting prevalence and mean *z*‐scores (HAZ) are estimated using National Family Health Surveys (NFHS) waves 1992–1993 and 2005–2006. Poverty HCR is obtained from the Government of India (2009). PCNSDP and PCDE (in 2004–2005 prices) are sourced from the Reserve Bank of India (https://rbi.org.in/).

HCR, headcount ratio; PCNSDP, per capita net state domestic product; PCDE, per capita state developmental expenditure.

**Figure 1:**
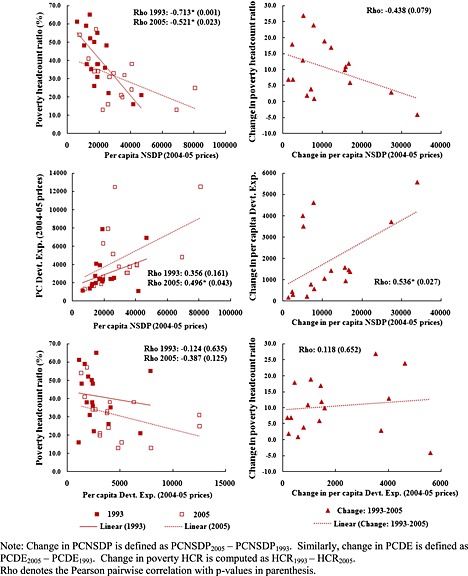
Correlation between levels and changes in per capita net state domestic product (NSDP), per capita developmental expenditure and poverty headcount ratio, Indian States 1992–1993 and 2005–2006. Change in per capita net state domestic product (PCNSDP) is defined as PCNSDP_2005_–PCNSDP_1993_. Similarly, change in per capita state developmental expenditure (PCDE) is defined as PCDE_2005_–PCDE_1993_. Change in poverty headcount ratio (HCR) is computed as HCR_1993_–HCR_2005_. Rho denotes the Pearson pairwise correlation with *P*‐values in parenthesis.

The correlation between PCNSDP and PCDE in 1993 and 2005 was 0.356 (*P*‐value 0.161) and 0.496 (0.043), respectively (Fig. 1). During the same time period, the strength of the correlation between PCNSDP and poverty HCR was −0.713 (0.001) and −0.521 (0.023), respectively. However, the correlation between PCDE and poverty HCR was statistically insignificant [correlation 1993: −0.124 (0.635) and 2005: −0.387 (0.125)], and interestingly, there was no correlation across the changes in these three developmental variables either (right‐side panel of Fig. 1).

The levels of childhood stunting were very high across most of the Indian states, and there was no perceptible decline during 1990s (Table 1). In 1993, the magnitude of stunting ranged from 59.4% (57.2–61.6) in Bihar to 31.9% (27.0–36.8) in Manipur. Between 1993 and 2005, only marginal improvements in stunting prevalence are noted. The ratios of change in stunting prevalence to changes in the three developmental variables are also low. In 12 out of the 17 states, 1% increase in PCNSDP was associated with 0 to 0.2 percentage point reduction in stunting prevalence (Table S3). Nine out of 17 states had similar values for public development expenditure, and 11 out the 17 states had an increase of approximately 1 percentage point in stunting for a percentage point increase in poverty ratio.

### Macroeconomic growth, public developmental expenditure and poverty

Table 2 and Fig. 1 present the results from the analysis in levels as well change over time to determine if macroeconomic growth during the period resulted in increased PCDE and poverty reduction.

**Table 2:** Coefficient estimates for ecological association of developmental variables, Indian States 1992–1993 and 2005–2006

		Levels		Change in change	
Model	Dependent variable	PCNSDP	PCDE	PCNSDP	PCDE
1	PCDE	0.35**	–	0.26*	–
		(0.04)	–	(0.11)	–
2	HCR	−2.14**	–	−2.1	–
		(0.70)	–	(1.1)	–
3	HCR	–	−6.70**	–	1.16
		–	(1.40)	–	(2.52)

PCNSDP, per capita net state domestic product; PCDE, per capita state developmental expenditure; HCR, headcount ratio.

Ecological models: standard error of the coefficient is reported in (parenthesis). All the models include an intercept term. The levels analysis is based on 34 observations available from 17 states observed at two points of time (1993 and 2005). The change in change analysis is based on 17 observations from 17 states. PCNSDP is expressed in units of Rs. 5000, whereas PCDE is expressed in units of RS. 2000.

** *P* < 0.01 and

* *P* < 0.05.

#### State income and development expenditure

In case of levels (Table 2 model 1), the coefficient estimates for PCNSDP is 0.35 (0.27; 0.44) implying that an increase in PCNSDP by INR 5000 is associated with an increase of INR 700 in PCDE. In other words, one rupee increase in PCNSDP is associated with 0.14 rupee increase in PCDE. The middle panel in Fig. 1 provides the graphical representation of this correlation between the two variables, and the relationship seems to have strengthened both in magnitude and statistical significance, from 1993 [0.356 (0.161)] to 2005 [0.496 (0.043)]. Not surprisingly, this improvement in the relationship over time is captured in the change on change model (Table 2 right‐side panel) albeit the magnitude is smaller compared with the levels and also revealed in Fig. 1 (right‐side panel): 0.536 (0.027).

#### State income and poverty

Similarly, the results also reveal a statistically significant but small association between levels of PCNSDP and poverty HCR. The estimates from levels model show that an increase in PCNSDP by INR 5000 is associated with a reduction of 2.1 (−3.51; − 0.77) percentage points in HCR, while the change in change model reveals no considerable relationship (model 2 in Table 3). Also, this association seems to have weakened over the two time periods, which is represented in the top panel of Fig. 1: 1993 [−0.713 (0.001)] and 2005 [−0.521 (0.023)]. It is noteworthy to mention that the relationship between HCR and PCDE has not been significant either in levels or change over time.

**Table 3:** Coefficient estimates for ecological models for the association of stunting prevalence with developmental variables, Indian States 1992–1993 and 2005–2006

Ecological		Levels			Change in change		
Model	Dependent variable	PCNSDP	PCDE	HCR	PCNSDP	PCDE	HCR
1	Stunting prevalence	−1.92**	–	–	−0.87	–	–
		(0.45)	–	–	(0.69)	–	–
2	Stunting prevalence	–	−5.41**	–	–	1.39	–
		–	(0.89)	–	–	(1.47)	–
3	Stunting prevalence	–	–	0.55**	–	–	0.20
		–	–	(0.08)	–	–	(0.14)

PCNSDP, per capita net state domestic product; PCDE, per capita state developmental expenditure; HCR, headcount ratio.

Standard error of the coefficient are reported in (parenthesis).

** *P* < 0.01 and

* *P* < 0.05.

In sum, Table 2 and Fig. 1 indicate that while the association between PCNSDP and PCDE has strengthened in recent years (though modestly), it is the opposite for PCNSDP and HCR. This phenomenon is also reflected in the change in change models, where the relationship with PCNSDP is stronger for PCDE compared with HCR (Fig. 1).

**Figure 2:**
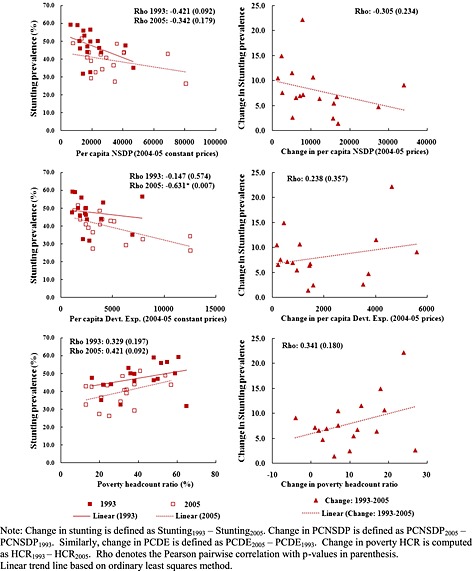
Correlation between levels and changes in early childhood stunting and key developmental indicators, Indian States 1992–1993 and 2005–2006. Change in stunting is defined as Stunting_1993_–Stunting_2005_. Change in per capita net state domestic product (PCNSDP) is defined as PCNSDP_2005_–PCNSDP_1993_. Similarly, change in per capita state developmental expenditure (PCDE) is defined as PCDE_2005_–PCDE_1993_. Change in poverty headcount ratio (HCR) is computed as HCR_1993_–HCR_2005_. Rho denotes the Pearson pairwise correlation with *P*‐values in parenthesis. Linear trend line based on ordinary least‐squares method.

### Public expenditure, poverty and stunting

#### Public expenditure and stunting

To test the association between PCDE and stunting, we undertake the following analysis in the same order: (1) employ ecological models – level on level and change on change and (2) multilevel modelling – unadjusted and adjusted (see Fig. 2). In the ecological model (Table 3), only in case of cross‐sectional regressions, we find a statistically significant association between PCDE and stunting. An increase of two thousand rupees in PCDE is associated with 5.4 (−7.15; −3.66) percentage point reduction in stunting prevalence (levels model 2), while there is no statistically significant relationship in the change on change model (right‐side panel), which is in line with the existing recent literature mentioned earlier. Similarly, the multilevel logistic model (Table 4 model 2) that adjusts for age and sex of the child as well as the survey year neither provides evidence for statistical relationship between the two variables. We arrive at similar conclusions even after adjusting for other individual and socioeconomic status (SES) correlates (right‐side panel).

**Table 4:** Odds ratio for multilevel models for the association of stunting prevalence with developmental variables, Indian States 1992–1993 and 2005–2006

Multilevel		Unadjusted Model (without SES variables)			Fully adjusted models (with SES variables)		
Model	Dependent variable	PCNSDP	PCDE	HCR	PCNSDP	PCDE	HCR
1	Stunting prevalence	1.059**	–	–	1.069**	–	–
		(1.02, 1.10)	–	–	(1.04, 1.11)	–	–
2	Stunting prevalence	–	1.121*	–	–	1.095	–
		–	(1.01, 1.25)	–	–	(0.98, 1.22)	–
3	Stunting prevalence	–	–	1.005	–	–	1.004
		–	–	(1.00, 1.01)	–	–	(1.00, 1.01)

Ecological models: standard error of the coefficient is reported in (parenthesis). All the models include an intercept term. The analysis is based on 34 observations available from 17 states observed at two points in time 1993 and 2005.

Multilevel models: 95% confidence interval for the odds ratios is reported in (parenthesis). All models include an intercept term. Models also adjust for age and sex of the child and survey year. The models with SES variables adjust for the following socio‐economic variables: birth order, maternal co‐residence, mother's age at child birth, maternal and partner education, social group, religion, wealth quintile and place of residence. The (unweighted) analysis is based on 37 256 pooled observations available from 17 states in National Family Health Surveys (NFHS) 1992–1993 and 2005–2006. PCNSDP is expressed as multiple of 5000, and PCDE is expressed as multiple of 2000.

PCNSDP, per capita net state domestic product; PCDE, per capita state developmental expenditure; HCR, headcount ratio;

SES, socioeconomic status.

** *P* < 0.01 and

* *P* < 0.05.

#### Poverty and stunting

We follow the same sequence as in PCDE by first analysing the relationship between HCR and stunting through (1) ecological models – levels and change on change and (2) multilevel models – unadjusted and adjusted and broadly find similar patterns. Model 3 in Table 3 (levels model) shows that one percentage point increase in HCR is associated with 0.55 (0.40; 0.70) percentage point higher prevalence of stunting, while in the change‐on‐change model, the relationship turns out to be insignificant. In the multilevel logistic models adjusted for age and sex of the child as well as the survey year, there is no evidence of relationship between HCR and stunting (Table 4, model 3). As with PCDE, we arrive at similar conclusions even after adjusting for other individual and SES correlates. Largely, the results from Tables 3 and 4 indicate that growth in PCNSDP or increments in PCDE or reductions in poverty did not necessarily translate to significant reductions in the prevalence of stunting.

As a part of sensitivity check, we also report the socio‐economic covariate‐specific odds ratio from the fully adjusted models for stunting in Table S6. The result shows that the associations between key socio‐economic variables and stunting outcomes are consistent and in expected direction: children from the highest wealth quintile were 51% less likely to be stunted [odds ratio 0.49 (0.44; 0.55)]. Similarly, we also find a gradient in the individual risk of stunting across levels of maternal and paternal education (Subramanyam *et al.* 2011). We also perform similar econometric analysis to examine the association of these developmental variables with two other indicators of child undernutrition *viz*. underweight and wasting and arrive at similar conclusions regarding the associations (Table S7–S8).

In sum, the results from our analysis suggest that although there is an association among levels of PCNSDP, PCDE and HCR, the change‐on‐change analysis clearly indicates that macroeconomic growth did not necessarily translate to substantial increments in PCDE or huge reductions in poverty incidence. Also, the results indicate that public developmental spending expenditure or poverty, in turn, had no significant influence on the prevalence of stunting (see Fig. 2). Interestingly, the ecological analysis (Table S10 and S11) using NFHS 2005–2006 and RSOC 2013–2014 data emerges with similar conclusions regarding the association between child undernutrition and developmental variables (also see Figures S5–S8). However, due to unavailability of individual unit‐level data from the RSOC survey as well as lack of comparable ecological data from the NFHS 1992–1993, we are unable to draw any further conclusions regarding these associations in recent years.

## Discussion

While it is generally believed that macroeconomic growth, through poverty alleviation and increased public investments in developmental services, can help reduce incidence of stunting (Haddad *et al.* 2003; Black *et al.* 2008; UNICEF 2013; Smith & Haddad 2015), our study results confirm that macroeconomic growth in Indian states did not necessarily translate into increased public development expenditure or substantial reduction in poverty between 1992 and 2005. At the same time, we did not find a robust relationship between changes in public development expenditure and reductions in stunting or reductions in aggregate poverty and stunting either. In other words, the channel through which macroeconomic growth is generally expected to positively influence stunting has been ineffective perhaps owing to low spillover effect from macroeconomic growth to public development expenditure and poverty reduction.

It is plausible that our results in some measure might be influenced by certain data constraints. First, because of unavailability of information on household incomes, we used socio‐economic ranking as a proxy, yet data on income could provide insights on the direct impact of macroeconomic growth on household incomes and consequently on child stunting, although our findings are robust even in parsimonious models with no consideration of other variables. Second, owing to data inconsistencies and unavailability, we could not include all states of India in our analysis – but this elimination does not induce any kind of selection bias as our criteria of exclusion was not with respect to any specific pattern of income or undernutrition. Third, we rely on measurement of macroeconomic growth, public developmental spending and poverty incidence at two distinct points in times. While there is no reason to expect major fluctuations in the trend, a panel dataset would be ideal to capture the inter‐temporal effects of growth, poverty and developmental spending on child stunting better tracking the same units of observations over time. Finally, it is worthwhile to note that public development expenditure as well as state per capita income only serves as a reasonable proxy for determinants such as investments in health and sanitation and overall household incomes. However, it is plausible that some of the investments and changes related with households may not be well captured through these proxy determinants. This also implies that the respective regression coefficients of these variables are perhaps not completely immune to any kind of attenuation bias. Relatedly, the explanatory variables are also likely to be measured with error that could attenuate the association.

Interestingly, recent findings from RSOC (2013–2014) indicate a decline in prevalence of stunting (children below 5 years) from 48.0% in 2005–2006 (NFHS‐3) to 38.8% in RSOC 2013–2014 (UNICEF and Government of India 2015). In this regard, the ecological analysis using NFHS 2005–2006 and RSOC 2013–2014 suggests possible improvement in the association between anthropometric failure and developmental variables although this could not be confirmed from the graphical analysis (S5–S8). Nevertheless, it may be noted that there have been some important changes in the policy environment in India post‐2005–2006 with increased public investment on developmental programmes such as the National Rural Health Mission, National Rural Employment Guarantee Act, etc. Hence, with new individual level data on child undernutrition and other socio‐economic covariates, it would be necessary to revisit the dynamics of the relationship.

Overall, the results from our analysis unravel a few worrisome aspects related to nutritional health and development in India. First is the concern regarding low levels of PCDE to PCNSDP ratio, particularly in states with high burden of stunting (Figure S5) combined with disparities in quality and efficiency of developmental spending across the states (Varadharajan *et al.* 2013). In most Indian states, unfavourable political economy coupled with institutional inefficiencies act as impediments for socio‐economic public investments and attenuates the effectiveness of direct interventions aimed at nutritional health. The noted exceptions are Kerala and Tamil Nadu, where the political economy environment promotes development of policy frameworks to support interventions, and the quantity and quality of public spending in social sectors positively influence human development (Dreze & Sen 1989; Mehrotra 2006; Muraleedharan *et al.* 2011). For most other states, while the numbers may not always suggest unhealthy development expenditure to growth ratio, whether they translate to improved socio‐economic outcomes is debatable. The prominent examples are the universal programmes for food supply (Public Distribution System) and nutritional supplementation programme (Integrated Child Development Services and Mid‐day School Meal Program) that are fraught with distributional leakages and inefficiencies at each tier and agency level (Lokshin *et al.* 2005; Khera 2011; Shukla 2014) and thus impede the poor from reaping the benefits of the programmes. Clearly, such areas offer scope for achieving greater efficiency in developmental spending by eliminating institutional bottlenecks and strengthening programme implementation (Dreze and Sen 2013).

High incidence of poverty and slow pace of poverty reduction are other areas of concern for India. It is noted that there has been no substantial reduction in poverty in India between 1993 and 2005. The all‐India poverty HCR (as per official statistics) was 45.3% for 1993–1994 and 37.2% for 2004–2005, implying a 0.8 percentage point decline per year on average (Figure S3). However, the reduction in poverty was negligible in states such as Bihar or Uttar Pradesh, which have high prevalence of undernutrition. Even the reduction in incidence of poverty was lower in few high‐growth states such as Gujarat (Table 1). Arguably, the official estimates are perhaps gross underestimates of reality, a fact borne out by the growing public outcry regarding official estimates of poverty in India (Subramanian 2011). For instance, in 2004–2005, the poverty HCR based on the Lakdawala methodology was 27.5%, whereas it was 37.2% based on the Tendulkar methodology (Government of India 2014a, 2014b). Adding to these inconsistencies, the official poverty estimation in India continues to be based on methodologies rooted in caloric norms and focuses less on competing non‐food items essential to ensure basic capabilities and functioning. In other words, the official poverty line itself is set too low, whereas a pragmatic assessment would yield poverty thresholds that would reject the official conclusions regarding poverty reductions in India (Subramanian 2011; Government of India 2009). Hence, states that already have a poor undernutrition base are doubly disadvantaged. Given such complexities, it is no surprise that official estimates of poverty reductions in India have not displayed any systematic association with stunting.

At the same time, an equally disturbing feature in the growth process is its limited engagement with rural economy, particularly agriculture – a sector that performed sub‐optimally during the 1990s (Bhalla & Singh 2009). The issue is further aggravated with growing disparities in agricultural productivity across cultivating households and poor agriculture‐nutrition linkage in India (Dev 2012). In fact, India experienced worrisome trends in per capita calorie consumption during 1990s and early 2000s (Deaton & Dreze, 2009; Patnaik 2004), and the share of household expenditure was more on non‐food items (including fuel), which is misaligned with a growth process that could facilitate faster reduction in undernutrition (Basu & Basole 2012).

Furthermore, there is no evidence of macroeconomic growth being equalizing or pro‐poor in India (Jayraj & Subramanian 2012; Suryanarayana 2008; Kohli 2006; Sen and Himanshu 2004). Sectoral imbalances in economic growth have also had adverse impact on marginalized social groups. For example, the tribal populations that reside in remote geographical regions do not benefit much from the growth process and continue to share a higher burden of nutritional deprivation (53.9% stunting, 27.6% wasting and 54.5% underweight in 2005–2006, IIPS Macro‐International 2007). With several distributional issues and supply side bottlenecks around growth, it might be plausible to observe a lagged impact of growth on stunting, but it may require a longer period of high and sustained economic growth (Haddad *et al.* 2003).

In conclusion, a quantum leap in developmental spending and ‘inclusive’ macroeconomic growth is necessary to achieve a positive impact on stunting in India. Also, the PCDE has to be directed or invested in improving certain proximate determinants of stunting such as maternal and child care, food security, water and sanitation (Smith & Haddad 2015). Such focused investments further call for easing out the supply side bottlenecks of the existing institutional set‐up with greater transparency and efficiency, which could promote child health and nutrition in India.

## Source of Funding

No direct financial support or funding was obtained to conduct this study. WJ is supported by the Lown Scholars Program of the T.H. Chan Harvard School of Public Health. However, the Program had no role in study design, data collection and analysis, decision to publish, or preparation of the manuscript.

## Conflicts of interest

The authors declare that they have no conflicts of interest.

## Contributions

SVS conceptualized the study. WJ and RR contributed to conceptualization and WJ led the data analysis, interpretation and writing of the manuscript. RR and SVS contributed to data interpretation and writing.

## Supporting information

Supporting info item
