# Supplementary material for: Understanding the null‐to‐small association between increased macroeconomic growth and reducing child undernutrition in India: role of development expenditures and poverty alleviation
Source: Matern Child Nutr. 2016 May 17;12(Suppl 1):196–209. doi: 10.1111/mcn.12256 (PMC5084736; doi:10.1111/mcn.12256)
Supplement: Supplementary file 1 — Supporting info item [file MCN-12-196-s001.docx]

**Supplemental Tables and Figures**

**Table S1: Descriptive statistics of socioeconomic variables used in the analysis, NFHS 1992-93 and 2005-06**

| **Variables** | **N** | **Mean** | **SD** | **Minimum** | **Maximum** |
| --- | --- | --- | --- | --- | --- |
| Stunting proportion (0/1) | 37256 | 0.46 | 0.50 | 0 | 1 |
| Underweight proportion (0/1) | 38817 | 0.41 | 0.49 | 0 | 1 |
| Wasting proportion (0/1) | 37073 | 0.21 | 0.41 | 0 | 1 |
| Per capita NSDP in INR | 44909 | 21106.57 | 13844.13 | 6257 | 80844 |
| Per capita Devt. Exp in INR | 44909 | 2980.97 | 2386.18 | 1112 | 12544 |
| Poverty headcount ratio (%) | 44909 | 39.11 | 14.38 | 7 | 67 |
| Year 2005-06 | 44909 | 0.44 | 0.50 | 0 | 1 |
| Child's age12-23 months | 44909 | 0.34 | 0.47 | 0 | 1 |
| Child's age 23-35 months | 44909 | 0.32 | 0.47 | 0 | 1 |
| Male child | 44909 | 0.52 | 0.50 | 0 | 1 |
| Birth order 2 | 44909 | 0.26 | 0.44 | 0 | 1 |
| Birth order 3 | 44909 | 0.17 | 0.38 | 0 | 1 |
| Birth order 4 and above | 44909 | 0.28 | 0.45 | 0 | 1 |
| Maternal age at birth 13-16 years | 44909 | 0.02 | 0.14 | 0 | 1 |
| Maternal age at birth 20-24 years | 44909 | 0.39 | 0.49 | 0 | 1 |
| Maternal age at birth 25-30 years | 44909 | 0.28 | 0.45 | 0 | 1 |
| Maternal age at birth 30 years and above | 44909 | 0.15 | 0.36 | 0 | 1 |
| Mother stays with husband | 44909 | 0.89 | 0.32 | 0 | 1 |
| Partner has primary education | 44909 | 0.19 | 0.39 | 0 | 1 |
| Partner has secondary education | 44909 | 0.42 | 0.49 | 0 | 1 |
| Partner has higher education | 44909 | 0.11 | 0.31 | 0 | 1 |
| Mother has primary education | 44909 | 0.13 | 0.34 | 0 | 1 |
| Mother has secondary education | 44909 | 0.29 | 0.45 | 0 | 1 |
| Mother has higher education | 44909 | 0.06 | 0.24 | 0 | 1 |
| Scheduled caste | 44909 | 0.15 | 0.36 | 0 | 1 |
| Scheduled tribe | 44909 | 0.11 | 0.31 | 0 | 1 |
| Islam | 44909 | 0.38 | 0.49 | 0 | 1 |
| Other religion | 44909 | 0.19 | 0.40 | 0 | 1 |
| Second wealth quintile | 44909 | 0.18 | 0.38 | 0 | 1 |
| Third wealth quintile | 44909 | 0.20 | 0.40 | 0 | 1 |
| Fourth wealth quintile | 44909 | 0.23 | 0.42 | 0 | 1 |
| Highest wealth quintile | 44909 | 0.22 | 0.41 | 0 | 1 |
| Rural areas | 44909 | 0.68 | 0.47 | 0 | 1 |

**Table S2: State-wise prevalence of underweight and wasting, Indian States 1992-93 and 2005-06**

| **States** | **Underweight (%)** | | **Wasting (%)** | |
| --- | --- | --- | --- | --- |
|  | ***1993*** | ***2005*** | ***1993*** | ***2005*** |
| Arunachal Pradesh | 33.0 | 32.8 | 16.4 | 16.2 |
| Assam | 44.4 | 36.2 | 14.3 | 17.2 |
| Bihar | 60.8 | 55.2 | 32.1 | 33.7 |
| Delhi | 36.7 | 27.5 | 15.9 | 16.9 |
| Goa | 31.0 | 21.6 | 18.1 | 13.7 |
| Gujarat | 44.3 | 41.8 | 24.2 | 20.3 |
| Haryana | 31.6 | 38.6 | 8.0 | 22.9 |
| Jammu & Kashmir | 37.7 | 25.7 | 18.8 | 18.7 |
| Karnataka | 47.6 | 35.0 | 24.1 | 19.1 |
| Kerala | 22.2 | 23.8 | 13.8 | 15.5 |
| Maharashtra | 46.9 | 35.0 | 27.9 | 17.6 |
| Manipur | 20.3 | 19.3 | 10.0 | 11.3 |
| Meghalaya | 38.8 | 53.6 | 17.7 | 32.1 |
| Odisha | 50.6 | 40.6 | 28.5 | 23.7 |
| Punjab | 40.6 | 23.6 | 21.0 | 10.1 |
| Rajasthan | 45.5 | 38.6 | 24.4 | 22.7 |
| Uttar Pradesh | 54.7 | 42.7 | 23.4 | 19.8 |

Note: Stunting prevalence and mean z-scores are estimated using NFHS waves 1992-93 and 2005-06. See Table 1 for stunting and information related to developmental indicators (Poverty HCR, per capita NSDP and per capita developmental spending).

**Table S3: State-wise ratio of percentage point reduction in undernutrition outcomes with percentage change in developmental indicators, 1993-94 and 2004-05**

| **States** | **Ratio for stunting with** | | | **Ratio for underweight** | | | **Ratio for wasting** | | |
| --- | --- | --- | --- | --- | --- | --- | --- | --- | --- |
|  | **PCNSDP** | **PCDE** | **HCR** | **PCNSDP** | **PCDE** | **HCR** | **PCNSDP** | **PCDE** | **HCR** |
| Arunachal Pradesh | 0.5 | 0.4 | 0.9 | 0.0 | 0.0 | 0.0 | 0.0 | 0.0 | 0.0 |
| Assam | 0.9 | 0.7 | 0.8 | 0.5 | 0.4 | 0.5 | -0.2 | -0.1 | -0.2 |
| Bihar | 0.4 | 0.7 | 1.5 | 0.2 | 0.4 | 0.8 | -0.1 | -0.1 | -0.2 |
| Delhi | 0.1 | 0.0 | 1.6 | 0.1 | 0.0 | 3.1 | 0.0 | 0.0 | -0.3 |
| Goa | 0.1 | 0.1 | -2.3 | 0.1 | 0.1 | -2.4 | 0.1 | 0.1 | -1.1 |
| Gujarat | 0.0 | 0.0 | 0.2 | 0.0 | 0.0 | 0.4 | 0.0 | 0.1 | 0.7 |
| Haryana | 0.1 | 0.1 | 0.6 | -0.1 | -0.1 | -0.6 | -0.2 | -0.2 | -1.2 |
| Jammu & Kashmir | 0.4 | 0.1 | 0.9 | 0.4 | 0.1 | 0.9 | 0.0 | 0.0 | 0.0 |
| Karnataka | 0.1 | 0.1 | 0.4 | 0.2 | 0.2 | 0.7 | 0.1 | 0.1 | 0.3 |
| Kerala | 0.1 | 0.1 | 0.5 | 0.0 | 0.0 | -0.1 | 0.0 | 0.0 | -0.2 |
| Maharashtra | 0.0 | 0.0 | 0.3 | 0.2 | 0.2 | 1.2 | 0.2 | 0.2 | 1.0 |
| Manipur | 0.1 | 0.0 | 0.1 | 0.0 | 0.0 | 0.0 | 0.0 | 0.0 | 0.0 |
| Meghalaya | 0.2 | 0.4 | 0.6 | -0.2 | -0.6 | -0.8 | -0.2 | -0.6 | -0.8 |
| Odisha | 0.1 | 0.5 | 3.3 | 0.2 | 0.8 | 5.0 | 0.1 | 0.4 | 2.4 |
| Punjab | 0.2 | 0.3 | 7.2 | 0.6 | 0.8 | 17.0 | 0.4 | 0.5 | 10.9 |
| Rajasthan | 0.1 | 0.2 | 1.8 | 0.1 | 0.2 | 1.7 | 0.0 | 0.0 | 0.4 |
| Uttar Pradesh | 0.3 | 0.3 | 1.1 | 0.5 | 0.5 | 1.7 | 0.1 | 0.2 | 0.5 |

Note: The ratios for PCNSDP and PCDE are computed as follows: Percentage point change in undernutrition prevalence divided by percentage increase in PCNSDP or PCDE. The ratio with respect to poverty HCR is computed as: Percentage point change in undernutrition prevalence divided by percentage point reduction in poverty HCR

**Table S4: Coefficient estimates for ecological models for the association of underweight and wasting prevalence with developmental variables, Indian States 1992-93 and 2005-06**

| **Ecological** |  | **Levels** | | | **Change in change** | | |
| --- | --- | --- | --- | --- | --- | --- | --- |
| **Model** | **Dependent variable** | **PCNSDP** | **PCDE** | **HCR** | **PCNSDP** | **PCDE** | **HCR** |
| 1 | Underweight prevalence | -1.46** | - | - | -0.24 | - | - |
|  |  | [0.51] | - | - | [1.17] | - | - |
| 2 | Underweight prevalence | - | -3.32** | - | - | -0.01 | - |
|  |  | - | [1.18] | - | - | [2.42] | - |
| 3 | Underweight prevalence | - | - | 0.16 | - | - | -0.44 |
|  |  | - | - | [0.12] | - | - | [0.22] |
| 4 | Wasting prevalence | -0.15 | - | - | 0.10 | - | - |
|  |  | [0.40] | - | - | [1.00] | - | - |
| 5 | Wasting prevalence | - | -0.30 | - | - | 0.11 | - |
|  |  | - | [0.92] | - | - | [2.07] | - |
| 6 | Wasting prevalence | - | - | -0.11 | - | - | -0.34 |
|  |  | - | - | [0.08] | - | - | [0.19] |

Note: ** p<0.01 and * p<0.05

Ecological models: Standard error of the coefficient are reported in [parenthesis]. All the models include an intercept term. The analysis is based on 34 observations available from 17 states observed at two points of time (1993 and 2005).

**Table S5: Odds ratio for multilevel models for the association of underweight and wasting prevalence with developmental variables, Indian States 1992-93 and 2005-06**

| **Multilevel** |  | **Unadjusted Model (without SES controls)** | | | **Fully adjusted model (with SES controls)** | | |
| --- | --- | --- | --- | --- | --- | --- | --- |
| **Model** | **Dependent variable** | **PCNSDP** | **PCDE** | **HCR** | **PCNSDP** | **PCDE** | **HCR** |
| 1 | Underweight prevalence | 1.047* | - | - | 1.058** | - | - |
|  |  | [1.01, 1.08] | - | - | [1.02, 1.09] | - | - |
| 2 | Underweight prevalence | - | 1.035 | - | - | 1.001 | - |
|  |  | - | [0.93, 1.16] | - | - | [0.90, 1.11] | - |
| 3 | Underweight prevalence | - | - | 0.983** | - | - | 0.982** |
|  |  | - | - | [0.97, 0.99] | - | - | [0.97, 0.99] |
| 4 | Wasting prevalence | 1.002 | - | - | 1.008 | - | - |
|  |  | [0.96, 1.04] | - | - | [0.97, 1.05] | - | - |
| 5 | Wasting prevalence | - | 0.979 | - | - | 0.974 | - |
|  |  | - | [0.86, 1.11] | - | - | [0.86, 1.11] | - |
| 6 | Wasting prevalence | - | - | 0.977** | - | - | 0.975** |
|  |  | - | - | [0.97, 0.99] | - | - | [0.97, 0.99] |

Note: ** p<0.01 and * p<0.05

Multilevel models: 95% confidence interval for the odds ratios are reported in [parenthesis]. All models include an intercept term. All models adjust for age and sex of the child and survey year. The models with socioeconomic status (SES) controls adjusts for the following socioeconomic variables: birth order, maternal co-residence, mother’s age at child birth, maternal and partner education, social group, religion, wealth quintile and place of residence. The (unweighted) analysis is based on pooled observations (38817 and 37073 for underweight and wasting, respectively) available from 17 states in NFHS 1992-93 and 2005-06. PCNSDP is expressed as multiple of 5000 and PCDE are expressed as multiple of 2000.

**Table S6: Fully adjusted multilevel logistic models for the association between early childhood stunting and developmental variables, Indian States 1992-93 and 2005-06**

| **Individual risk of stunting** | **Model 1** | | **Model 2** | | **Model 3** | |
| --- | --- | --- | --- | --- | --- | --- |
|  | **OR** | **se** | **OR** | **se** | **OR** | **se** |
| Per capita NSDP (5000s) | 1.070*** | 0.018 |  |  |  |  |
| Per capita Devt. Exp (2000s) |  |  | 1.095* | 0.058 |  |  |
| Poverty headcount ratio (%) |  |  |  |  | 1.004 | 0.004 |
| Year 1992-93# |  |  |  |  |  |  |
| Year 2005-06 | 0.619*** | 0.033 | 0.667*** | 0.035 | 0.732*** | 0.043 |
| Child's age 0-12 months# |  |  |  |  |  |  |
| Child's age12-23 months | 3.037*** | 0.086 | 3.037*** | 0.086 | 3.039*** | 0.086 |
| Child's age 23-35 months | 4.201*** | 0.122 | 4.198*** | 0.122 | 4.199*** | 0.122 |
| Female child# |  |  |  |  |  |  |
| Male child | 1.151*** | 0.026 | 1.151*** | 0.026 | 1.151*** | 0.026 |
| Birth order 1# |  |  |  |  |  |  |
| Birth order 2 | 1.114*** | 0.036 | 1.112*** | 0.036 | 1.112*** | 0.036 |
| Birth order 3 | 1.148*** | 0.043 | 1.145*** | 0.043 | 1.144*** | 0.043 |
| Birth order 4 and above | 1.273*** | 0.050 | 1.271*** | 0.050 | 1.267*** | 0.050 |
| Maternal age at birth 17-19 years# |  |  |  |  |  |  |
| Maternal age at birth 13-16 years | 1.376*** | 0.123 | 1.373*** | 0.123 | 1.373*** | 0.123 |
| Maternal age at birth 20-24 years | 0.902*** | 0.031 | 0.903*** | 0.031 | 0.902*** | 0.031 |
| Maternal age at birth 25-30 years | 0.793*** | 0.031 | 0.794*** | 0.031 | 0.795*** | 0.031 |
| Maternal age at birth 30 years and above | 0.802*** | 0.038 | 0.805*** | 0.038 | 0.807*** | 0.038 |
| Stays separate# |  |  |  |  |  |  |
| Mother stays with husband | 1.013 | 0.038 | 1.013 | 0.038 | 1.016 | 0.038 |
| Partner is illiterate# |  |  |  |  |  |  |
| Partner has primary education | 0.953 | 0.034 | 0.950 | 0.034 | 0.949 | 0.034 |
| Partner has secondary education | 0.863*** | 0.029 | 0.864*** | 0.029 | 0.864*** | 0.029 |
| Partner has higher education | 0.703*** | 0.038 | 0.703*** | 0.038 | 0.704*** | 0.038 |
| Mother is illiterate# |  |  |  |  |  |  |
| Mother has primary education | 0.951 | 0.035 | 0.948 | 0.035 | 0.946 | 0.035 |
| Mother has secondary education | 0.779*** | 0.028 | 0.780*** | 0.028 | 0.781*** | 0.028 |
| Mother has higher education | 0.617*** | 0.045 | 0.620*** | 0.045 | 0.620*** | 0.045 |
| Other social group# |  |  |  |  |  |  |
| Scheduled caste | 1.171*** | 0.040 | 1.174*** | 0.040 | 1.173*** | 0.040 |
| Scheduled tribe | 1.080 | 0.052 | 1.081 | 0.052 | 1.078 | 0.052 |
| Hinduism# |  |  |  |  |  |  |
| Islam | 1.029 | 0.044 | 1.032 | 0.044 | 1.022 | 0.044 |
| Other religion | 0.969 | 0.047 | 0.964 | 0.047 | 0.960 | 0.046 |
| Lowest wealth quintile# |  |  |  |  |  |  |
| Second wealth quintile | 0.908** | 0.036 | 0.909** | 0.036 | 0.911** | 0.036 |
| Third wealth quintile | 0.837*** | 0.034 | 0.839*** | 0.034 | 0.841*** | 0.034 |
| Fourth wealth quintile | 0.733*** | 0.033 | 0.733*** | 0.033 | 0.734*** | 0.033 |
| Highest wealth quintile | 0.488*** | 0.027 | 0.489*** | 0.027 | 0.487*** | 0.027 |
| Urban areas# |  |  |  |  |  |  |
| Rural areas | 0.961 | 0.033 | 0.963 | 0.033 | 0.963 | 0.033 |

Note: ***p<0.01, ** p<0.05 and * p<0.1

# denotes reference category for the concerned variables. All models use state fixed effects model and include an intercept term. The models with socioeconomic status (SES) controls adjusts for the following socioeconomic variables: birth order, maternal co-residence, mother’s age at child birth, maternal and partner education, social group, religion, wealth quintile and place of residence. The (unweighted) analysis is based on pooled observations (37256, 38817 and 37073 for stunting, underweight and wasting, respectively) available from 17 states in NFHS 1992-93 and 2005-06. PCNSDP is expressed as multiple of 5000 and PCDE are expressed as multiple of 2000.

**Table S7: Fully adjusted multilevel logistic models for the association between early childhood underweight and developmental variables, Indian States 1992-93 and 2005-06**

| **Individual risk of underweight** | **Model 1** | | **Model 2** | | **Model 3** | |
| --- | --- | --- | --- | --- | --- | --- |
|  | **OR** | **se** | **OR** | **se** | **OR** | **se** |
| Per capita NSDP (5000s) | 1.058*** | 0.018 |  |  |  |  |
| Per capita Devt. Exp (2000s) |  |  | 1.001 | 0.055 |  |  |
| Poverty headcount ratio (%) |  |  |  |  | 0.982*** | 0.004 |
| Year 1992-93# |  |  |  |  |  |  |
| Year 2005-06 | 0.712*** | 0.037 | 0.789*** | 0.041 | 0.662*** | 0.039 |
| Child's age 0-12 months# |  |  |  |  |  |  |
| Child's age12-23 months | 1.621*** | 0.044 | 1.622*** | 0.044 | 1.622*** | 0.044 |
| Child's age 23-35 months | 1.813*** | 0.050 | 1.814*** | 0.050 | 1.814*** | 0.050 |
| Female child# |  |  |  |  |  |  |
| Male child | 1.175*** | 0.026 | 1.174*** | 0.026 | 1.174*** | 0.026 |
| Birth order 1# |  |  |  |  |  |  |
| Birth order 2 | 1.094*** | 0.035 | 1.093*** | 0.035 | 1.092*** | 0.035 |
| Birth order 3 | 1.114*** | 0.041 | 1.111*** | 0.041 | 1.111*** | 0.041 |
| Birth order 4 and above | 1.216*** | 0.047 | 1.212*** | 0.046 | 1.215*** | 0.047 |
| Maternal age at birth 17-19 years# |  |  |  |  |  |  |
| Maternal age at birth 13-16 years | 1.299*** | 0.109 | 1.297*** | 0.109 | 1.299*** | 0.109 |
| Maternal age at birth 20-24 years | 0.933** | 0.031 | 0.934** | 0.031 | 0.934** | 0.031 |
| Maternal age at birth 25-30 years | 0.897*** | 0.034 | 0.898*** | 0.034 | 0.897*** | 0.034 |
| Maternal age at birth 30 years and above | 0.903** | 0.042 | 0.906** | 0.042 | 0.903** | 0.042 |
| Stays separate# |  |  |  |  |  |  |
| Mother stays with husband | 1.011 | 0.037 | 1.013 | 0.037 | 1.011 | 0.037 |
| Partner is illiterate# |  |  |  |  |  |  |
| Partner has primary education | 0.984 | 0.033 | 0.981 | 0.033 | 0.982 | 0.033 |
| Partner has secondary education | 0.853*** | 0.027 | 0.854*** | 0.027 | 0.855*** | 0.027 |
| Partner has higher education | 0.749*** | 0.041 | 0.749*** | 0.041 | 0.749*** | 0.041 |
| Mother is illiterate# |  |  |  |  |  |  |
| Mother has primary education | 0.925** | 0.033 | 0.921** | 0.033 | 0.921** | 0.033 |
| Mother has secondary education | 0.752*** | 0.027 | 0.754*** | 0.027 | 0.752*** | 0.027 |
| Mother has higher education | 0.494*** | 0.038 | 0.496*** | 0.038 | 0.495*** | 0.038 |
| Other social group# |  |  |  |  |  |  |
| Scheduled caste | 1.222*** | 0.040 | 1.224*** | 0.040 | 1.227*** | 0.040 |
| Scheduled tribe | 1.107** | 0.052 | 1.107** | 0.052 | 1.115** | 0.052 |
| Hinduism# |  |  |  |  |  |  |
| Islam | 0.948 | 0.039 | 0.948 | 0.039 | 0.970 | 0.041 |
| Other religion | 0.885** | 0.043 | 0.880*** | 0.042 | 0.889** | 0.043 |
| Lowest wealth quintile# |  |  |  |  |  |  |
| Second wealth quintile | 0.942 | 0.035 | 0.944 | 0.035 | 0.940* | 0.035 |
| Third wealth quintile | 0.792*** | 0.031 | 0.795*** | 0.031 | 0.795*** | 0.031 |
| Fourth wealth quintile | 0.670*** | 0.029 | 0.671*** | 0.029 | 0.671*** | 0.029 |
| Highest wealth quintile | 0.438*** | 0.024 | 0.438*** | 0.024 | 0.439*** | 0.024 |
| Urban areas# |  |  |  |  |  |  |
| Rural areas | 0.950 | 0.033 | 0.951 | 0.033 | 0.951 | 0.033 |

Note: ***p<0.01, ** p<0.05 and * p<0.1

# denotes reference category for the concerned variables. All models use state fixed effects model and include an intercept term. The models with socioeconomic status (SES) controls adjusts for the following socioeconomic variables: birth order, maternal co-residence, mother’s age at child birth, maternal and partner education, social group, religion, wealth quintile and place of residence. The (unweighted) analysis is based on pooled observations (37256, 38817 and 37073 for stunting, underweight and wasting, respectively) available from 17 states in NFHS 1992-93 and 2005-06. PCNSDP is expressed as multiple of 5000 and PCDE are expressed as multiple of 2000.

**Table S8: Fully adjusted multilevel logistic models for the association between early childhood wasting and developmental variables, Indian States 1992-93 and 2005-06**

| **Individual risk of wasting** | **Model 1** | | **Model 2** | | **Model 3** | |
| --- | --- | --- | --- | --- | --- | --- |
|  | **OR** | **se** | **OR** | **se** | **OR** | **se** |
| Per capita NSDP (5000s) | 1.008 | 0.020 |  |  |  |  |
| Per capita Devt. Exp (2000s) |  |  | 0.974 | 0.063 |  |  |
| Poverty headcount ratio (%) |  |  |  |  | 0.975*** | 0.005 |
| Year 1992-93# |  |  |  |  |  |  |
| Year 2005-06 | 0.963 | 0.059 | 0.992 | 0.060 | 0.777*** | 0.054 |
| Child's age 0-12 months# |  |  |  |  |  |  |
| Child's age12-23 months | 0.860*** | 0.027 | 0.860*** | 0.027 | 0.859*** | 0.027 |
| Child's age 23-35 months | 0.550*** | 0.019 | 0.550*** | 0.019 | 0.550*** | 0.019 |
| Female child# |  |  |  |  |  |  |
| Male child | 1.166*** | 0.031 | 1.166*** | 0.031 | 1.166*** | 0.031 |
| Birth order 1# |  |  |  |  |  |  |
| Birth order 2 | 1.127*** | 0.043 | 1.126*** | 0.043 | 1.126*** | 0.043 |
| Birth order 3 | 1.131*** | 0.050 | 1.130*** | 0.050 | 1.131*** | 0.050 |
| Birth order 4 and above | 1.181*** | 0.054 | 1.180*** | 0.054 | 1.185*** | 0.055 |
| Maternal age at birth 17-19 years# |  |  |  |  |  |  |
| Maternal age at birth 13-16 years | 1.121 | 0.113 | 1.120 | 0.113 | 1.124 | 0.113 |
| Maternal age at birth 20-24 years | 1.046 | 0.043 | 1.046 | 0.043 | 1.047 | 0.043 |
| Maternal age at birth 25-30 years | 1.011 | 0.047 | 1.012 | 0.047 | 1.009 | 0.047 |
| Maternal age at birth 30 years and above | 1.025 | 0.056 | 1.026 | 0.057 | 1.020 | 0.056 |
| Stays separate# |  |  |  |  |  |  |
| Mother stays with husband | 0.966 | 0.041 | 0.967 | 0.041 | 0.964 | 0.041 |
| Partner is illiterate# |  |  |  |  |  |  |
| Partner has primary education | 1.002 | 0.040 | 1.001 | 0.040 | 1.003 | 0.040 |
| Partner has secondary education | 0.917** | 0.035 | 0.917** | 0.035 | 0.918** | 0.035 |
| Partner has higher education | 0.845** | 0.056 | 0.845** | 0.056 | 0.845** | 0.056 |
| Mother is illiterate# |  |  |  |  |  |  |
| Mother has primary education | 0.974 | 0.042 | 0.973 | 0.042 | 0.974 | 0.042 |
| Mother has secondary education | 0.849*** | 0.037 | 0.849*** | 0.037 | 0.846*** | 0.037 |
| Mother has higher education | 0.756*** | 0.068 | 0.757*** | 0.068 | 0.754*** | 0.068 |
| Other social group# |  |  |  |  |  |  |
| Scheduled caste | 1.123*** | 0.043 | 1.123*** | 0.043 | 1.126*** | 0.044 |
| Scheduled tribe | 1.200*** | 0.064 | 1.200*** | 0.064 | 1.212*** | 0.065 |
| Hinduism# |  |  |  |  |  |  |
| Islam | 0.981 | 0.048 | 0.980 | 0.048 | 1.011 | 0.050 |
| Other religion | 0.950 | 0.055 | 0.948 | 0.055 | 0.960 | 0.055 |
| Lowest wealth quintile# |  |  |  |  |  |  |
| Second wealth quintile | 0.968 | 0.042 | 0.969 | 0.042 | 0.963 | 0.041 |
| Third wealth quintile | 0.863*** | 0.039 | 0.864*** | 0.039 | 0.862*** | 0.039 |
| Fourth wealth quintile | 0.797*** | 0.041 | 0.797*** | 0.041 | 0.797*** | 0.041 |
| Highest wealth quintile | 0.652*** | 0.043 | 0.652*** | 0.043 | 0.654*** | 0.043 |
| Urban areas# |  |  |  |  |  |  |
| Rural areas | 1.007 | 0.041 | 1.007 | 0.041 | 1.007 | 0.041 |

Note: ***p<0.01, ** p<0.05 and * p<0.1

# denotes reference category for the concerned variables. All models use state fixed effects model and include an intercept term. The models with socioeconomic status (SES) controls adjusts for the following socioeconomic variables: birth order, maternal co-residence, mother’s age at child birth, maternal and partner education, social group, religion, wealth quintile and place of residence. The (unweighted) analysis is based on pooled observations (37256, 38817 and 37073 for stunting, underweight and wasting, respectively) available from 17 states in NFHS 1992-93 and 2005-06. PCNSDP is expressed as multiple of 5000 and PCDE are expressed as multiple of 2000.

**Table S9: Prevalence of undernutrition among children below age 5, NFHS 2005-06 and RSOC 2013-14**

|  | Stunting | | Wasting | | Underweight | |
| --- | --- | --- | --- | --- | --- | --- |
| **State** | 2005-06 | 2013-14 | 2005-06 | 2013-14 | 2005-06 | 2013-14 |
| Arunachal Pradesh | 43.3 | 28.4 | 15.3 | 17 | 32.5 | 24.6 |
| Assam | 46.5 | 40.6 | 13.7 | 9.7 | 36.4 | 22.2 |
| Bihar | 55.6 | 49.4 | 27.1 | 13.1 | 55.9 | 37.1 |
| Delhi | 42.2 | 29.2 | 15.4 | 14.2 | 26.1 | 19.4 |
| Goa | 25.6 | 21.3 | 14.1 | 15.4 | 25 | 16.2 |
| Gujarat | 51.7 | 41.8 | 18.7 | 18.7 | 44.6 | 33.5 |
| Haryana | 45.7 | 36.5 | 19.1 | 8.8 | 39.6 | 22.7 |
| Jammu & Kashmir | 35 | 31.9 | 14.8 | 7.1 | 25.6 | 15.6 |
| Karnataka | 43.7 | 34.7 | 17.6 | 17.5 | 37.6 | 29 |
| Kerala | 24.5 | 19.5 | 15.9 | 15.5 | 22.9 | 18.5 |
| Maharashtra | 46.3 | 35.4 | 16.5 | 18.6 | 37 | 25.2 |
| Manipur | 35.6 | 33.2 | 9 | 7.1 | 22.1 | 14.1 |
| Meghalaya | 55.1 | 42.9 | 30.7 | 13.1 | 48.8 | 30.9 |
| Orissa | 45 | 38.2 | 19.5 | 18.3 | 40.7 | 34.4 |
| Punjab | 36.7 | 30.5 | 9.2 | 8.7 | 24.9 | 16.1 |
| Rajasthan | 43.7 | 36.5 | 20.4 | 14.2 | 39.9 | 31.5 |
| Uttar Pradesh | 56.8 | 50.6 | 14.8 | 10 | 42.4 | 34.5 |

Note: 2005-06 data is based on National Family Health Survey 2005-06 (IIPS & Macro International 2007)

2013-14 data is based on Rapid Survey on Children 2013-14 conducted by UNICEF and Government of India.

**Table S10: Coefficient estimates for ecological association of developmental variables, Indian States 2004-05 and 2011-12**

|  |  | **Levels** | | **Change in change** | |
| --- | --- | --- | --- | --- | --- |
| **Model** | **Dependent variable** | **PCNSDP** | **PCDE** | **PCNSDP** | **PCDE** |
| 1 | PCDE | 0.58** | - | -0.36 | - |
|  |  | [0.18] | - | [0.26] | - |
| 2 | HCR | -2.18** | - | 0.52 | - |
|  |  | [0.39] | - | [0.72] | - |
| 3 | HCR | - | -1.24** | - | -1.48* |
|  |  | - | [0.41] | - | [0.57] |

Note: ** p<0.01 and * p<0.05

Ecological models: Standard error of the coefficient are reported in [parenthesis]. All the models include an intercept term. The levels analysis is based on 34 observations available from 17 states observed at two points of time. The change in change analysis is based on 17 observations from 17 states. PCNSDP is expressed in units of Rs. 5000 whereas PCDE is expressed in units of RS. 2000.

**Table S11: Coefficient estimates for ecological models for the association of undernutrition prevalence with developmental variables, Indian States 2005-06 and 2013-14**

| **Ecological** |  | **Levels** | | | **Change in change** | | |
| --- | --- | --- | --- | --- | --- | --- | --- |
| **Model** | **Dependent variable** | **PCNSDP** | **PCDE** | **HCR** | **PCNSDP** | **PCDE** | **HCR** |
| 1 | Stunting prevalence | -1.47** | - | - | 0.27 | - | - |
|  |  | [0.22] | - | - | [0.30] | - | - |
| 2 | Stunting prevalence | - | -1.30** | - | - | 0.23 | - |
|  |  | - | [0.18] | - | - | [0.28] | - |
| 3 | Stunting prevalence | - | - | 0.43** | - | - | -0.87 |
|  |  | - | - | [0.08] | - | - | [0.11] |
| 4 | Underweight prevalence | -1.70** | - | - | -0.27 | - | - |
|  |  | [0.32] | - | - | [0.37] | - | - |
| 5 | Underweight prevalence | - | -1.67** | - | - | 0.11 | - |
|  |  | - | [0.24] | - | - | [0.34] | - |
| 6 | Underweight prevalence | - | - | 0.61** | - | - | 0.01 |
|  |  | - | - | [0.09] | - | - | [0.13] |
| 7 | Wasting prevalence | -0.37 | - | - | -0.71 | - | - |
|  |  | [0.25] | - | - | [0.47] | - | - |
| 8 | Wasting prevalence | - | -0.70** | - | - | 0.29 | - |
|  |  | - | [0.19] | - | - | [0.46] | - |
| 9 | Wasting prevalence | - | - | 0.21* | - | - | -0.05 |
|  |  | - | - | [0.08] | - | - | [0.18] |

Note: ** p<0.01 and * p<0.05

**Figure S1: Prevalence of stunting, underweight and wasting and association with per capita NSDP, Indian States 1992-93 and 2005-06**


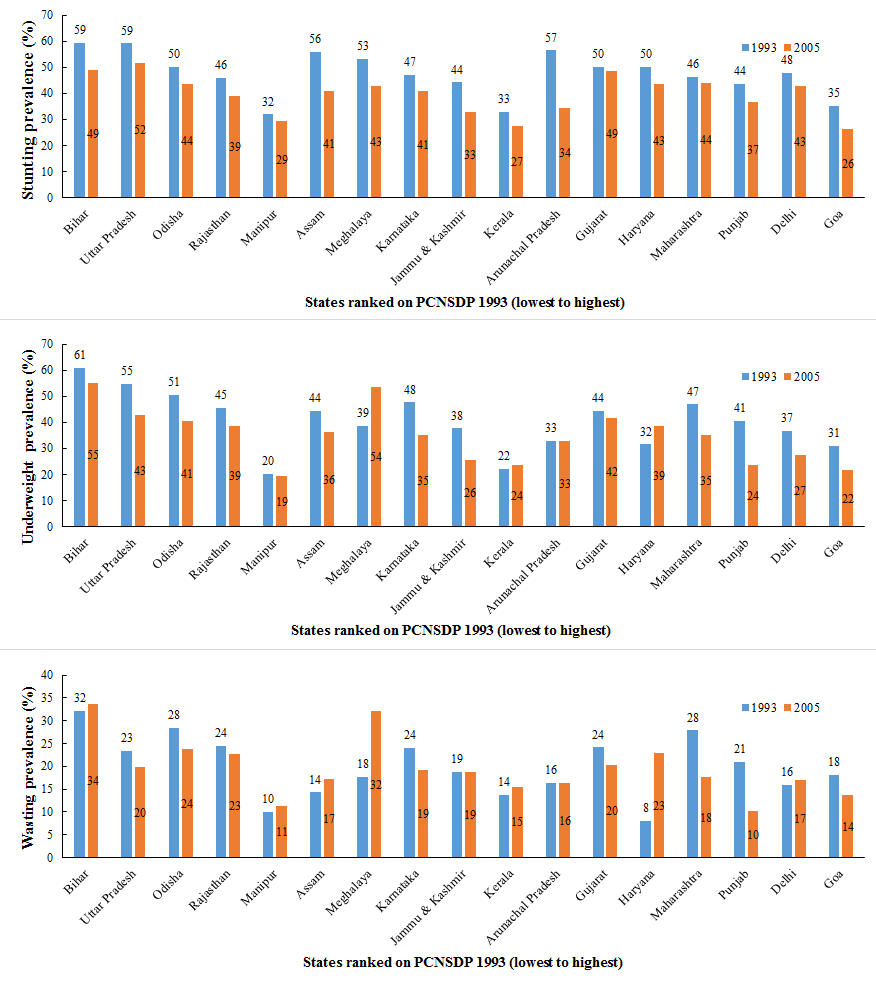


**Figure S2: Correlation between levels and changes in early childhood underweight and key developmental indicators, Indian States 1992-93 and 2005-06**


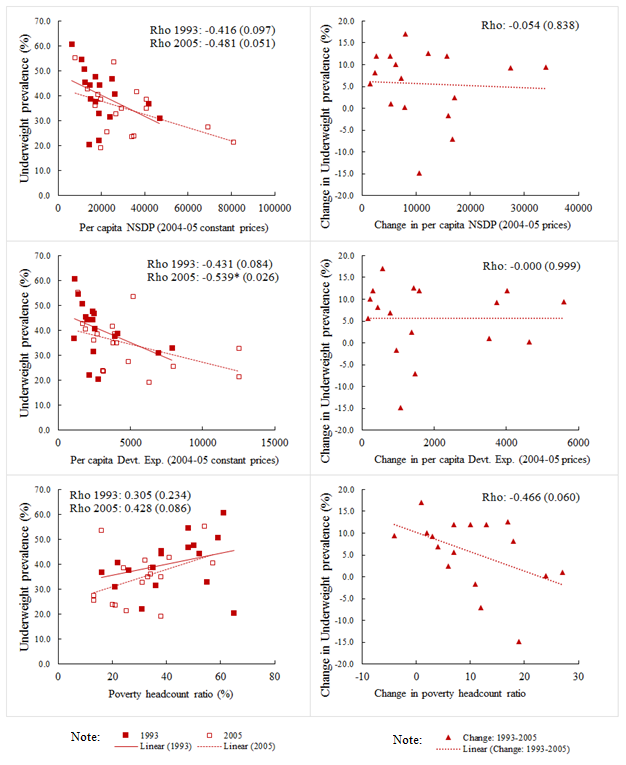


Note: Change in underweight is defined as Underweight_1993_ – Underweight_2005_. Change in PCNSDP is defined as PCNSDP_2005_ – PCNSDP_1993_. Similarly, change in PCDE is defined as PCDE_2005_ – PCDE_1993_. Change in poverty HCR is computed as HCR_1993_ – HCR_2005_. Rho denotes the Pearson pairwise correlation with p-values in parenthesis.

Linear trend line based on ordinary least squares method.

**Figure S3: Correlation between levels and changes in early childhood wasting and key developmental indicators, Indian States 1992-93 and 2005-06**


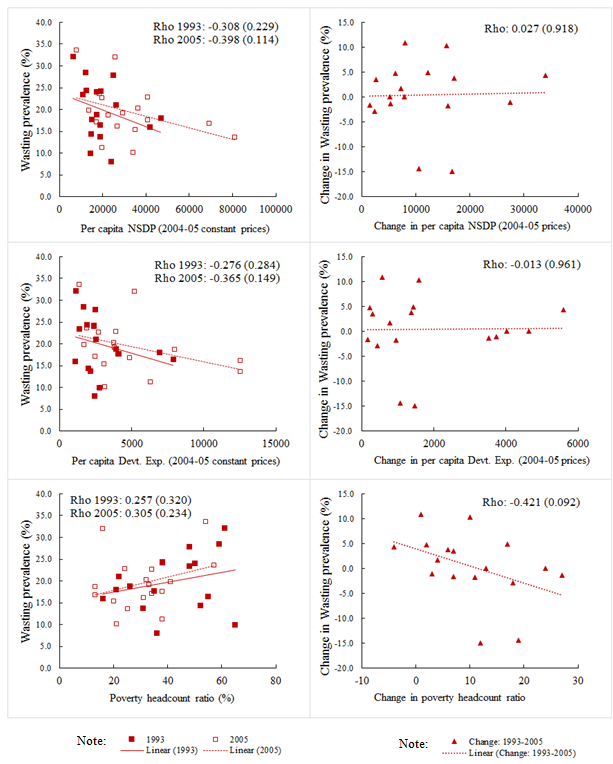


Note: Change in wasting is defined as Wasting_1993_ – Wasting_2005_. Change in PCNSDP is defined as PCNSDP_2005_ – PCNSDP_1993_. Similarly, change in PCDE is defined as PCDE_2005_ – PCDE_1993_. Change in poverty HCR is computed as HCR_1993_ – HCR_2005_. Rho denotes the Pearson pairwise correlation with p-values in parenthesis.

Linear trend line based on ordinary least squares method.

**Figure S4: Per capita developmental expenditure as a percentage share of per capita NSDP, Indian States 1992-93 and 2005-06**


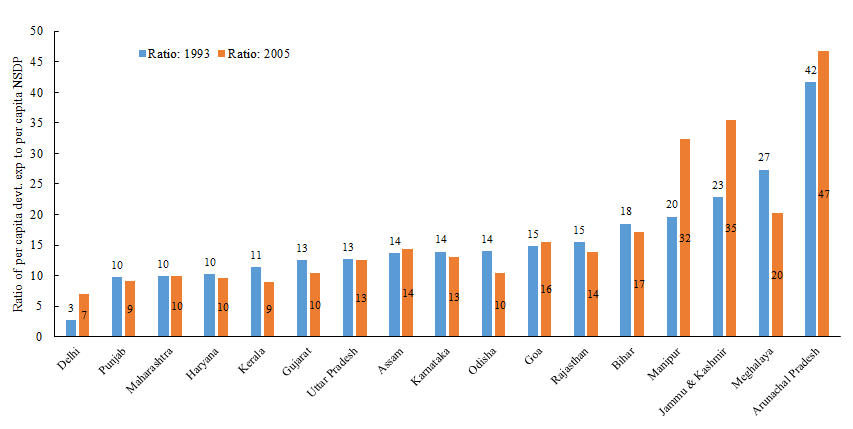


**Figure S5: Correlation between levels and changes in per capita NSDP, per capita developmental expenditure and poverty headcount ratio, Indian States 2004-05 and 2011-12**


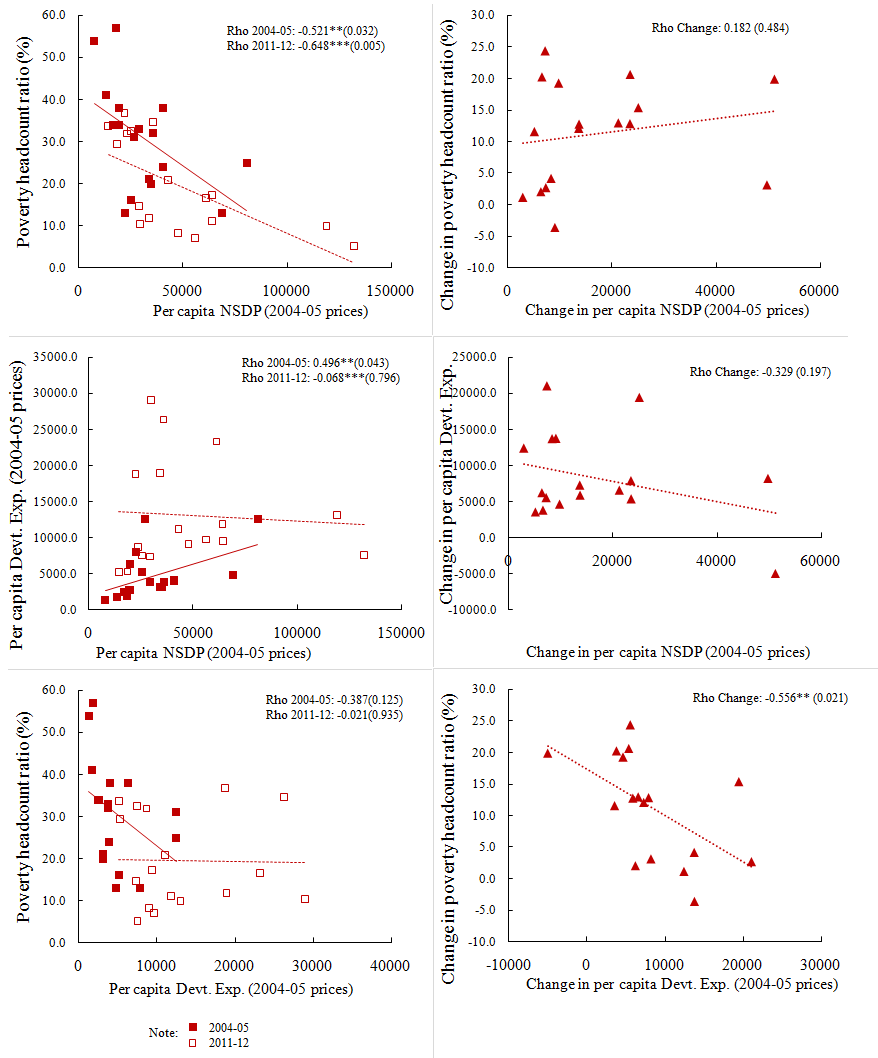


Note: Rho denotes the Pearson pairwise correlation with p-values in parenthesis.

**Figure S6: Correlation between levels and changes in child (aged below 5 years) stunting and key developmental indicators, Indian States 2005-06 and 2013-14**


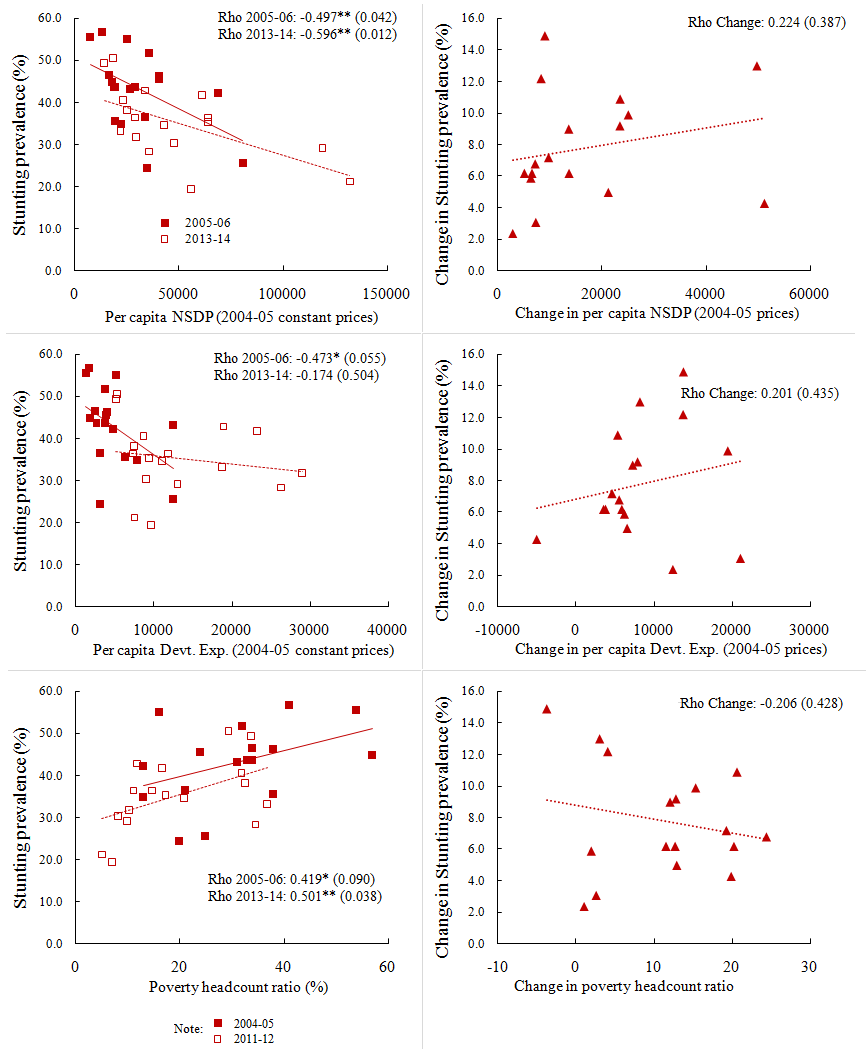


Note: Rho denotes the Pearson pairwise correlation with p-values in parenthesis.

**Figure S7: Correlation between levels and changes in child (aged below 5 years) underweight and key developmental indicators, Indian States 2005-06 and 2013-14**


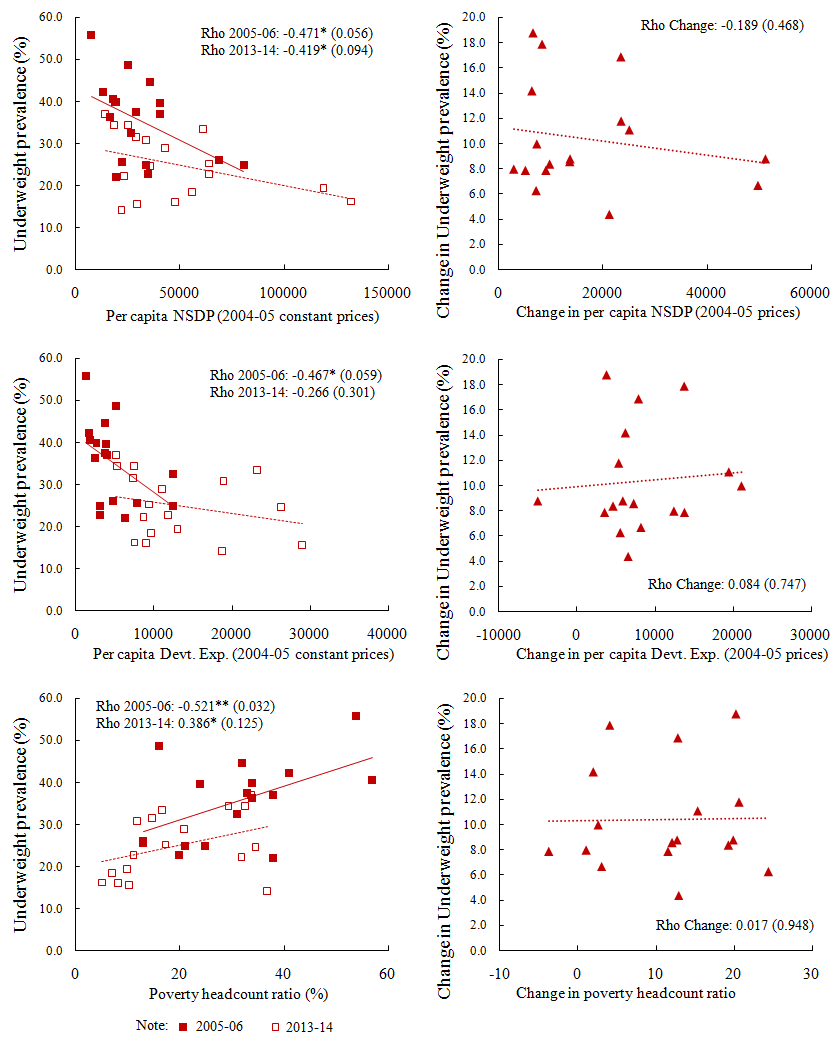


Note: Rho denotes the Pearson pairwise correlation with p-values in parenthesis.

**Figure S8: Correlation between levels and changes in child (aged below 5 years) wasting and key developmental indicators, Indian States 2005-06 and 2013-14**


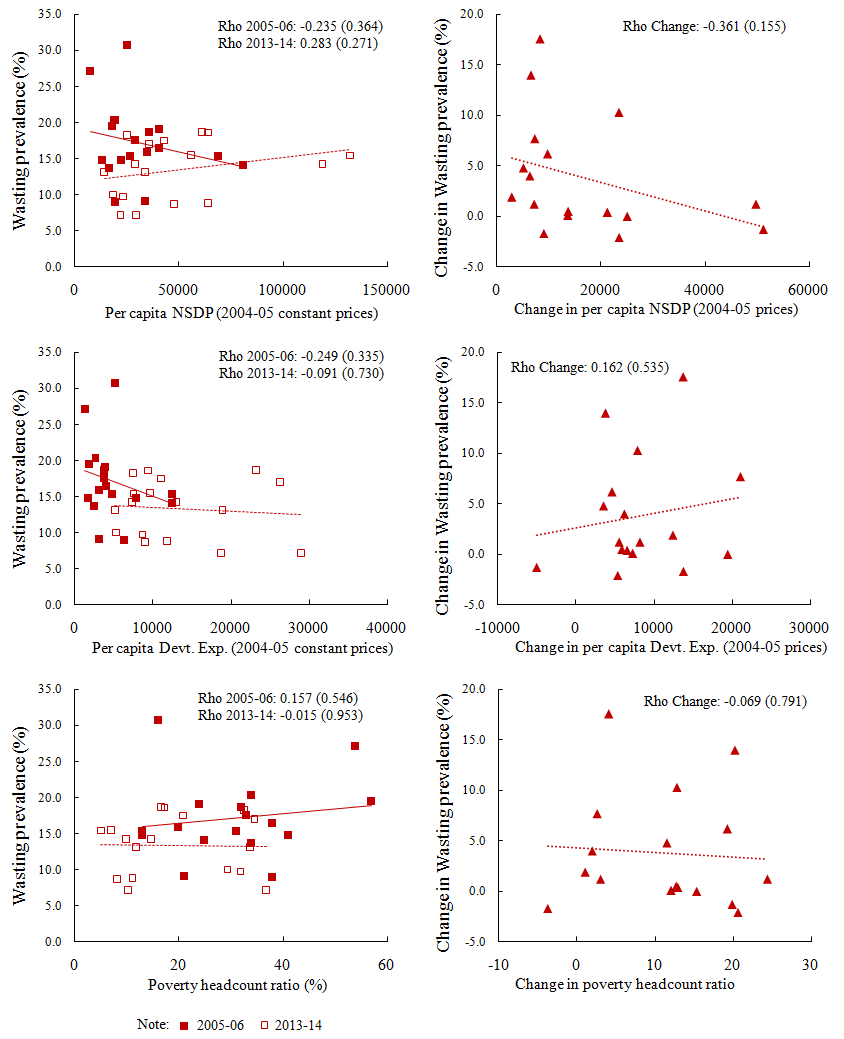


Note: Rho denotes the Pearson pairwise correlation with p-values in parenthesis.
